# Health staff experiences with the implementation of early essential newborn care guidelines in Da Nang municipality and Quang Nam province in Viet Nam

**DOI:** 10.1186/s12913-020-05449-2

**Published:** 2020-06-26

**Authors:** Marianne S. Morseth, Tuan T. Nguyen, Malene Skui, Laura Terragni, Quang V. Ngo, Ha T. T. Vu, Roger Mathisen, Sigrun Henjum

**Affiliations:** 1 Department of Nursing and Health Promotion, Oslo Metropolitan University, Oslo, Norway; 2 Alive & Thrive Southeast Asia, Hanoi, FHI 360 Viet Nam; 3 Center for Disease Control, Da Nang Department of Health, Da Nang, Viet Nam

**Keywords:** Early essential newborn care, Early initiation of breastfeeding, Health staff experiences, Viet Nam

## Abstract

**Background:**

The World Health Organization (WHO) recommends early essential newborn care (EENC) – The First Embrace – as a simple lifesaving procedure for newborns. The successful implementation of EENC at scale requires an understanding of health staff experiences, including facilitators, barriers, and local adaptations of EENC. This study aims to gain insight into health staff experiences with implementation of EENC guidelines after participation in training and coaching initiatives in Da Nang municipality and Quang Nam province in Viet Nam.

**Methods:**

In each province/municipality, we randomly selected one hospital from the following categories: public provincial/municipal hospital, public district hospital, and private hospital. We conducted in-depth interviews with 19 hospital staff (11 midwives, 5 doctors and 3 health managers) and two trainers during 7 days between September and October 2017. We used deductive/inductive thematic analysis to generate themes.

**Results:**

The health staff reported improved staff and mother satisfaction, and health benefits for both mothers and newborns after implementing EENC. Facilitators to successful implementation were management support for resource allocation and collaboration across departments, and creative demand generation. Barriers included staff shortage, skepticism about the new protocols and practices and challenges translating knowledge and skills from trainings and coaching into practice.

**Conclusions:**

After implementing EENC, through training and coaching using the WHO approach, health staff reported improved staff and mother satisfaction as well as health benefits for both mothers and newborns. An approach to develop competencies, with a focus on practical training and coaching, should be promoted to form, reinforce and sustain recommended EENC practices among health staff.

## Background

The United Nations’ Sustainable Development Goals aim to end preventable deaths of newborns and children under 5 years of age by 2030, including reducing the neonatal mortality rate from 19 to 12 deaths per 1000 live births, and lowering the under-5 mortality rate from 43 to 25 deaths per 1000 live births [1, 2]. Meeting these targets is challenging worldwide due to insufficient progress in low- and lower-middle-incomes member states, especially among disadvantaged groups [3–5]. In the world, mortality during the neonatal period accounted for nearly half of all deaths among children under 5 and two-thirds of death in the first year of life [2, 6, 7]. Thus, it is critical, to devise effective and affordable interventions for newborns [4, 8].

One of the efforts initiated by the World Health Organization (WHO) to reduce early neonatal mortality is the promotion of Early Essential Newborn Care (EENC), which ensures quality of care during childbirth and during the first 24 h after [8]. These guidelines present a set of steps consisting of immediate and thorough drying of the newborn, immediate skin-to-skin contact, delayed umbilical cord clamping until pulsations stop, and early initiation of breastfeeding (EIBF) [7]. The practices help to 1) transfer warmth, placental blood, and protective bacteria from the mother to the newborn, 2) promote a natural bond between mother and child, and 3) feed the newborn colostrum, which provides essential nutrients, antibodies, and immune cells to protect against disease [9]. After successful implementation in eight prioritized member states in the regions, the WHO Regional Office for the Western Pacific Region issued a comprehensive package for EENC training and coaching [10] and is rolling out the guidelines beyond the region [11]. In each member state, the process includes 1) updating monitoring and evaluation data to check status, track progress, and identify action to strengthen implementation, 2) reinforcing EENC policy, planning and coordination, and 3) putting into practice EENC and providing coaching and quality improvement [11, 12].

In 2016, experts from WHO supported the Ministry of Health in Viet Nam to develop national technical guidelines based on WHO principles for essential maternal and newborn care in 2014 [13] and for cesarean births [14]. Using WHO training materials and approaches, national trainers, with support from WHO experts, provided training and coaching to provincial trainers, who will facilitate the rollout of training and coaching to facilities that provide maternity and newborn services in their provinces and municipalities. The training and coaching were supported technically by WHO Viet Nam and the Ministry of Health and financially by the Ministry of Health and partners, including United Nations Children’s Fund (UNICEF) and Alive & Thrive (A&T). To date, most of the data on EENC implementation for Viet Nam was from the routine monitoring systems of Viet Nam Ministry of Health and biennial reviews [11, 12, 15]. To our knowledge, few peer-reviewed publications have reported health staff experiences with the implementation of EENC guidelines using a qualitative approach. We conducted this qualitative study to gain insight into health staff experiences with implementation of EENC guidelines after participation in the training and coaching initiatives in Da Nang municipality and Quang Nam province in Viet Nam.

## Methods

### Study context

Viet Nam is a lower-middle income country situated in Southeast Asia with a population of 95 million (34% in urban areas), a life expectancy at birth of 76 years, and a youth (15–24 years) literacy rate of 97%. Additional data on characteristics of Viet Nam are shown in Table 1. Viet Nam has high levels of antenatal care coverage, institutional births, births attended by a skilled birth attendant of (~ 95%) and postnatal health checks for newborns and mothers (~ 90%) [2]. Each year, among 1.6 million newborns, 19,000 die in the first 28 days of life, a rate which makes up ~ 70% of deaths among infants and 55% of deaths of children under 5 [2]. In 2016, the prevalence of cesarean births was 28% and the prevalence of early initiation of breastfeeding was 27% [2, 17]. Findings from WHO biennial reviews indicate that there is room for increased prolonged skin-to-skin contact and sustained skin-to-skin contact until the first breastfeeding in both term and preterm babies, especially in subnational hospitals [15].

**Table 1:** General characteristics of Viet Nam and studied area (in 2017)^a^

	Viet Nam	Da Nang	Quang Nam
Area (Km^2^)	331,230.80	1284.90	10,574.70
Average population (Thousand)	93,671.60	1064.10	1493.80
Population density (Person/km^2^)	283	828	141
Percentage of literate population	95.1	98.2	95.7
Crude birth rate	14.9	15.2	15.2
Crude death rate	6.8	6.3	8.7
Natural increase rate	8.1	8.9	6.5
Total fertility rate	2.04	1.81	2.26
Infant mortality rate	14.4	8.5	16.1
Under-five mortality rate	21.5	12.9	24.2

^a^Data from Viet Nam General Statistics Office [16]

To support the rollout of the EENC guidelines for vaginal births, A&T provided financial support and coordination efforts for training, coaching, supportive supervision, and monitoring using WHO approaches and materials, along with provincial or national trainers in seven provinces/municipalities. From December 2014 to March 2015, A&T supported local departments of health to roll out EENC guidelines for vaginal births: establishing EENC core teams, conducting facility-based coaching and supportive supervision, and collecting and using routine monitoring data for program improvement in all hospitals providing maternity and newborn services in the seven provinces. The participants at the A&T-supported training and coaching for vaginal births included 779 staff from 102 hospitals providing maternity and newborn services (Table 2). The participants were selected based on their involvement in maternity and newborn services, their position as influencers or managers, and their skills for follow-up training and coaching. The trained staff then became key stakeholders in planning and rolling out the guidelines in their own hospitals with the support of provincial/municipal facilitators and supervisors. A&T supported routine supportive supervision and coaching and data management to monitor and support service quality. The qualitative and quantitative data and information collected was reviewed during regular meetings for timely decision making. Learning exchanges were also arranged to share knowledge and experiences among managers and health workers for improved performance. In 2017 and 2018, A&T supported additional training and coaching to roll out EENC guidelines for cesarean births for 843 health staff in 78 hospitals across six provinces/municipalities (Table 2).

**Table 2:** Training and coaching supported by Alive & Thrive and EENC outcomes^a^

	A&T support provinces	Da Nang	Quang Nam
**Training and coaching**			
Guidelines for vaginal births			
Number of provinces	7		
Number of hospitals	102	13	23
Number of staff	779	185	92
Guidelines for cesarean births			
Number of provinces	6		
Number of hospitals	78	12	24
Number of staff	843	250	93
**Number of births with any early skin-to-skin**^**b**^			
Vaginal births, number (%)			
2015	54,000 (83%)	7000 (95%)	13,000 (94%)
2016	82,000 (94%)	9000 (96%)	16,000 (99%)
2017	81,000 (96%)	11,000 (96%)	16,000 (99%)
Cesarean births, number (%)			
2015	14,000 (35%)	9000 (75%)	4000 (52%)
2016	20,000 (37%)	13,000 (89%)	6000 (55%)
2017	30,000 (55%)	15,000 (97%)	9000 (84%)
Total, number (%)			
2015	68,000 (64%)	16,000 (83%)	17,000 (79%)
2016	102,000 (72%)	22,000 (92%)	22,000 (81%)
2017	111,000 (80%)	26,000 (97%)	25,000 (93%)

^a^ Data from monitoring system supported by Alive & Thrive

^b^ Data rounded to 1000

This qualitative study was conducted in Da Nang municipality and Quang Nam province in Viet Nam. As of 2017, Da Nang municipality had a population of 1 million and an infant mortality rate of 8.5 deaths per 1000 live births, while Quang Nam province had a population of 1.5 million and infant mortality rate of 16.1 deaths per 1000 live births (Table 1) [16]. EENC training and coaching was conducted to cover all hospitals providing maternity and newborn services, including both public and private hospitals (Table 2). In total, the number of health staff who directly received EENC training and coaching from national and provincial trainers were 185 for vaginal and 250 for cesarean births in Da Nang municipality, and 92 for vaginal births and 93 for cesarean births in Quang Nam province (Table 2). The reproductive health centers provide administration, technical guidance, training, coaching, and supportive supervision to all hospitals in the province/municipality. A national trainer, also a WHO expert in Da Nang Hospital for Women and Children, played an important role in the training, coaching, and implementation of maternal and newborn care, including EENC.

### Data collection

Prior to the data collection, a semi-structured interview guide was developed by staff at Oslo Metropolitan University and A&T (Supplementary file 1). The interview guide comprised questions in different categories and served as an aid to ensure that all necessary topics were covered during the interviews. Key categories were: 1) training in EENC, 2) changes after the training, 3) coverage of EENC in the hospital, 4) EENC implementation and breastfeeding practices, 5) EENC implementation and child health, and 6) EENC implementation and hospital policies and operations. The EENC trainer in Da Nang municipality provided inputs to this guide before interviews were conducted.

In this study, permission was granted to visit the province/municipality. In each province/municipality, data was collected from the provincial/municipal reproductive health centers and three hospitals that had been randomly selected from hospitals in these categories: a provincial/municipal public hospital, a district public hospital, and a private hospital. Staff at the provincial/municipal reproductive health centers communicated with the selected hospitals to seek their approval. All selected hospitals agreed to participate in the study.

During 7 days between September and October 2017, we conducted in-depth interviews with two provincial/municipal trainers and all 19 staff who had attended the training and coaching on EENC by provincial trainers and were in the hospitals during data collection (~ 10 staff were not in the hospitals for the interview). The communication between the interviewer (the third author) and the participants was facilitated by an English-Vietnamese interpreter. The interviewer was a female Norwegian master student in Public Health Nutrition. The interpreter was an A&T program associate who had a bachelor’s degree in foreign language (major in English and minor in French) and had worked with A&T for about 6 years. Each participant was interviewed in a quiet, private room in their workplace. Nineteen of 21 interviews were recorded, while two interviews were documented by handwritten notes because the participants did not grant consent to record. The average duration of the interviews was 30 min; all interviews ranged from 20 to 90 min.

### Transcription and analysis

The English content was transcribed within 15 days after the data collection by the third author. We used a deductive/inductive thematic analysis approach to identify relevant patterns within the data set [18, 19] and generate themes from initial questionnaires. Additional themes also emerged. A code book was developed, discussed, and agreed upon among the authors. The third author identified relevant parts from each transcribed interview, coded them, and aggregated them under main themes [20]. The authors reviewed statements from each theme to examine whether there were similarities or disagreements in the coding process, and to identify statements relevant to the purpose of this study.

## Results

Three main thematic areas and seven sub-themes emerged from the interviews. The first theme was experiences with the implementation of the program which included the sub-themes health staff and mother satisfaction and health benefits of implementing EENC. The second theme was facilitators to the implementation which included two sub-themes, management support and resources and creative demand generation. The third thematic area was barriers to successful implementation, which included three sub-themes: staff shortages during cesarean births, skepticism about new routines and challenges translating knowledge and skills from the training and coaching into improved practices.

### Experiences with the implementation

#### Health staff and mother satisfaction

All respondents reported that implementing EENC had been successful and beneficial.

*It brings happiness into my work (…*) *we see the benefits of EENC, and we see the actions of the baby, like seeking the breast. Everything is very natural, and I am very happy to see that.* (Midwife 2).

Some participants stated that the new routines of EENC improved mother satisfaction.

*(…*) *they cry – but this is happy tears. Because some, they have already given birth, but their other babies they did not receive EENC. When they receive EENC for this baby they are happy.* (Health manager 1)

Some participants mentioned that an increased demand for EENC in the community can shape providers’ practices.

*(…*) *I think for the common woman, most know about the skin-to-skin contact*-*method. So now, when they come to the hospital, the women even know that they have the right to have the skin-to-skin contact service, and women themselves can request it in the hospital. I heard stories about how when women come to the hospital, they ask, “do you provide skin-to-skin contact?” I think it is good to educate women well, so that they know their right (…*) (Health manager 2)

#### Health benefits of implementing EENC

Participants agreed on the health benefits of EENC. For example, one midwife shared her observations, noting fewer side effects, a faster recovery, and improved maternal experience.

(…) *Before we applied EENC in the hospital there was quite a lot of side effects that the mother faced after the cesarean births. The mother was very tired, or the body was shaking, or things like that. But when applying EENC, the mother focused on taking care of the baby. And they feel more excitement and feel better after cesarean births.* (Midwife 8)

Another respondent perceived that after the implementation of new routines, there were fewer sick infants.

*In the hospital, we do the skin-to-skin. This can be implemented widely in the operating room and birthing room. And in the neonatal unit, we see the benefits when there are fewer sick babies (…*) (Doctor 5).

Some respondents discussed how implementation of skin-to-skin contact had helped reduce the use of breastmilk substitutes in the hospital, because limiting unnecessary separation of the mother and newborn increased breastmilk production.

*(…*) *We see that the mothers are very happy. Although they are tired after giving birth, when they are with the baby, they forget all the tiredness and the milk production is better. They see the benefits of breastmilk (…*) (Midwife 2)

The importance of adapting EENC as the new routine was stated by many and were considered key to make the intervention succeed and ensure every mother and newborn receives the best care possible.

*I think that EENC is very necessary for both the mother and the baby. So, the doctors and the midwives here are very supportive to this. We try our best to do it as a routine, so that no cases will be missed out.* (Doctor 2).

### Facilitators

#### Management support and resources

The participants’ perception of resources and management support in the implementation of EENC were mostly favorable. One participant explained how the environment in the hospital changed after it was requested to implement EENC, and how close collaboration with other departments and support from the Hospital Director made implementation easier.

*(…*) *With the environment there’s a lot of change (…*) *Previously they had only 32 midwives, but now there are 48 midwives. They also collaborated with relevant departments, including the infection control department and the anesthesia department, which helped to work more smoothly. Also, close collaboration with other departments and support from the director of the hospital (…*) *To support EENC, the hospital provided more clothes to dry and cover the baby, which had not happened prior (…*) (Midwife 6)

The need for supportive leadership for implementation to succeed was noted by several participants. In addition, the importance of close supervision when implementing new routines was highlighted:

*There were differences when they first implemented this. During the dayshift, with supportive supervision from the head midwife and the head of the department, they did it very thoroughly. But then, during the night shift, due to the workload, and more patients, and without the supportive supervision and the on-site support from the head midwife and the head of the department, the midwife would shorten the duration on EENC – skin-to-skin (…*) *But now everything has become a routine activity, and they do it very well.* (Midwife 6).

#### Creative demand generation

Participants also described how private hospitals use EENC as a special service to attract expecting mothers.

*(…*) *There are some differences between implementing EENC in public and private hospitals. In a public hospital, implementing EENC is considered a part of the health worker’s role. In a private hospital, they could use EENC as a special service to attract more mothers to come for antenatal care and births. Specific add-on services of this [private] hospital include birth accompanied by family members and massage services for the mothers.* (Provincial trainer 1)

### Barriers

#### Health staff shortage during cesarean births

Although respondents did not experience any specific challenges in implementing EENC after vaginal births, some challenges were identified with regards to cesarean births. Firstly, it was perceived as problematic that some women were scheduled for cesarean births when it might not have been medically necessary.

*… That is everywhere in Viet Nam. Some families chose the date, the hour of birth.* (Health manager 2).

*(…*) *A problem in our country is that the cesarean birth rate now is very high, and we need urgent solutions, (…*) *one solution is educating the women about the benefits of vaginal birth, and the disadvantages of cesarean births (…*) (Doctor 5)

Secondly, the fact that cesarean births require more staff than vaginal births was repeatedly stated as a challenge.

*(…*) *When the mother is transferred to the post-operation room, they do not have enough staff to take care of the mother and the baby.* (Health manager 1)

*(…*) *one midwife will be in the operation room to support the EENC skin-to-skin. But then, if there are more patients here – it’s a birth – they will need to go back and support with the birth, so the skin-to-skin might be interrupted.* (Midwife 1)

The staff shortage was also perceived to be more severe during night shifts, which increased the challenges with cesarean births.

*(…*) *If we have many cesarean births at the same time, or during the night shift – there’s less staff to support. So, we cannot do skin-to-skin contact as expected (…*) (Doctor 3)

#### Skepticism about new routines

When asked about barriers among co-workers to implementing the new routines, several respondents mentioned skepticism, or even fear, about changing previous practices, especially at the initial stages of implementation.

*(…*) *For example, first they do the cleaning with the liquid (aspirating with the mucus) in the baby’s mouth and the nose – they need to take it out. And if they applied EENC they would not do that. And if they did not do that, they were afraid that the baby would choke – that it would create a problem for the baby. And the second thing is about the delayed cord clamping. Previously they would cut the cord, and they would apply some kind of alcohol onto the cord – to prevent infections. But now, with delayed cord clamping and EENC they will not apply any alcohol. So, they were afraid of infection. So that’s a thing they hesitated to do (…*) (Midwife 5)

*(…*) *the nurses and the midwives will assist with births, so they were afraid that the baby would fall when they put the baby on the mother’s chest (…*) (Doctor 1)

#### Challenges translating knowledge and skills from the training and coaching into improved practices

Although most participants expressed that EENC training and coaching was effective; and EENC implementation was successful after that, there is room for improvement. Some participants suggested to have more practice during the training.

*(…*) *They did not have much practice during the training course.* (Doctor 1)

Selected hospitals had challenges in translating knowledge and skills from the training and coaching into improved practices. A midwife reported that a few of the staff members got the chance to participate in training, which might have caused difficulties and disagreements when implementing new routines, because all staff may not have received the same information.

(…) *more staff should attend the training. Some of them attended the training, and they came back and re-trained others. But this is not as good as if all the staff attended the training from the provincial trainers.* (Midwife 7)

## Discussion

The health staff experienced many benefits from training and coaching on implementation of the new EENC guideline, including improved staff and mother satisfaction and health benefits for mothers and newborns. Patient satisfaction is closely linked to Quality of Care (QoC), which, according to the WHO, has two aspects: provision of evidence-based care by health workers and how care is experienced by patients [21]. In this study, the staff perceptions of clinical benefits and mothers’ positive responses to the new guidelines implies that QoC likely was perceived to have improved. The participants’ experience of mothers crying tears of happiness speaks to the rewards felt by the staff. The QoC model also emphasizes that improving QoC requires competent and motivated human resources as well as essential physical resources [21]. Targeting the competence of staff through coaching, as was conducted here, may be viewed as an individual-specific approach to improving quality of care. However, nursing practice is highly contextualized by the organization’s setting, intra-and inter-professional interaction, and multiple competing tasks [22]. This is also described in a study on health workers’ perceptions about what constitutes high-quality maternal and newborn care in rural Tanzania, where provision of care was perceived to be successful when things went as intended, when circumstances were predictable and the system was reliable [23]. At the same time, providing high-quality care likely motivates more women to seek health care [24], and a change in health-seeking behaviors. Mothers requesting EENC was observed by a few participants. A study from Finland on nurses’ experiences implementing new clinical guidelines found that patient awareness of the guidelines could help with successful implementation [25], similar to what was experienced here.

In our study, we found a challenge in the process of transitioning from learning to adopting and implementing EENC in the hospitals. The Normalization Process Theory (NPT) is a model that assists in explaining how new clinical guidelines become routinely embedded in health care practice [26]. According to NPT, a new routine is more likely to be sustained when staff understand the value, benefit, and importance of a new set of practices [22]. The staff experience of providing improved QoC might thus also have a positive influence on the implementation of EENC through positive effects on staff attitudes toward and personal commitment to the new guidelines [25]. For instance, in the study among birth attendants in Tanzania, observing a mother and baby to be in good condition was mentioned as a sign of having provided good quality care [23]. On the other hand, lack of outcome expectancy and motivation are found to be the main barriers to guideline implementation [27].

The participants’ emphasis on the importance of a conducive working environment resonates well with previous knowledge [25, 28, 29]. A qualitative study from Canada where administrators, nursing staff, and project managers were interviewed about factors influencing best practice guideline implementation found that leadership support, including provision of resources, was closely linked with positive staff attitudes and beliefs about the implementation [29]. Reports in this study that the number of midwives had been increased and that necessary equipment was in place after EENC had been implemented speaks to the management’s commitment to the intervention. In addition, participants expressed that collaboration with relevant departments, including Operation and Anesthesiology Units, Neonatal Care Unit, Post-Operative Wards, and Infection Control Department, was important. This again implies a coherent approach by management and is similar to the study from Canada where participants recognized interprofessional teamwork and collaboration as an important indicator for successful guideline implementation [29].

Although the staff spoke of few barriers to the implementation of EENC, some challenges, mainly related to cesarean births, were identified. Mainly, the participants talked about staff shortage as the primary challenge, which is a known barrier to successful improvement of health care practices [23, 28]. More specifically, participants shared experiences of having to leave mothers to themselves after cesarean births, interrupting skin-to-skin contact because of pressing work demands, especially during night shifts. Among birth attendants in Tanzania, the ability to stay close to the mother, both physically, to enable monitoring, and emotionally, was regarded as a sign of providing high-quality care. However, being the sole provider of maternal and newborn care in a health facility sometimes resulted in competing demands, which was perceived to result in untimely delivery of care [23]. According to NPT, the capability of nurses to implement a clinical guideline depends on its intrinsic workability and integration within the constraints of clinical practice [22]. Here, the new guidelines were perceived as saving staff resources. This was likely an important motivating factor for the staff to integrate EENC as the new routine.

Interruption of skin-to-skin contact after cesarean births, a concern which was shared by several participants, is problematic for various reasons. Firstly, skin-to-skin contact following cesarean births was reported by mothers in the US to have a calming effect, both for themselves and their newborns. Mothers also expressed that they felt empowered and experienced increased confidence in their maternal role, even in a highly medicalized setting [30]. This is echoed in our study firstly because mothers were calmer when receiving skin-to-skin contact after cesarean births, focusing more on their babies than on medical issues. Secondly, skin-to-skin contact is known to increase milk production [31]. A common reason for not performing EBF is a perception that the milk is insufficient [32, 33]. At the same time studies show that perceived milk insufficiency in most instances is alleviated by proper counselling by health staff [34]. In a study across 11 provinces in Viet Nam, the odds of pre-lacteal feeding (during the first 3 days after birth) after cesarean births compared to vaginal births was significantly greater [35]. Despite the great importance of EENC after cesarean births, staff shortages seemed to reduce QoC in this area. This was likely the main reason why several respondents considered it a challenge that many women could choose cesarean births. This is also recognized by the WHO in new guidance on non-clinical interventions specifically designed to reduce unnecessary cesarean births [36] and warning that medicalization of normal child birth may overburden front-line health workers [21].

One suggestion for improving learning outcomes was that all staff should be able to attend the coaching and training by national or provincial trainers. It was argued that it might be harder to convince health staff to follow guidelines and implement new routines if they had not received direct training. In a systematic review assessing successful interventions for promoting professional behavior change in healthcare work, Johnson and May (2015) observe that participants must have a clear impression that what they are asked to do makes sense and that their responses (changed practice) measure up to the expectations of external observers (i.e. audit and feedback) [37]. Further, in complex interventions, confidence in authoritative sources and agreement on criteria by which their credibility can be assessed is essential [26]. In a study from Kenya, one main barrier to guideline implementation was that not all health staff members were trained, resulting in a lack of knowledge and skills to apply the guidelines in general [38]. Although we found that it is not feasible to organize the training to all relevant staff at a hospital, there would be some improvements needed in some hospitals such as the selection of training participants (e.g., leadership, training skills) and ongoing coaching by provincial trainers and supervisors.

There were advantages to in-house training. For instance, there is the chance to influence management protocols in the case of disagreements with hospital authorities. This personal involvement will likely increase motivation for training and for the implementation to succeed [39]. At the same time, Johnson and May (2015) showed that interventions reinforcing modified peer group norms by emphasizing the expectations of an external reference group (in this case supervisors or management) offer the best chances of success [37]. As such, the staff perception that challenges due to skepticism and misconceptions faced at the beginning of the implementation now seemed to have ceased in part speaks to the importance of peer group norms for behavioral change.

Some participants expressed that there was not much practice during the training. Such impressions indicate that the training may not have adequately addressed the intent of the guidelines, which emphasize that at the end of on-site coaching, staff should demonstrate proficiency in new routines [10]. This conclusion is further supported by the literature, since meta-analyses show that successful behavior change interventions commonly incorporate new practice norms through experience [37], and resonate well with a new model developed by WHO for more on-the-job capacity development [10]. Learning experiences seem to be more lasting when participants are motivated to learn [40]. In this study, some health staff members who received training had many years of experience working with birth attendants. Adult learners are often more interested in practical learning than theory and want to learn exactly what is relevant to their work [40]. At the same time, although practice was not included in the initial training, the staff experienced the benefits and barriers of implementing EENC through their subsequent work. A recent study exploring facilitators and barriers to guideline implementation in Ethiopia indicated that continuous monitoring, evaluation, and mentorship were critical elements in the integration of the guideline into the system of the hospital [41].

The main advantage of the study was the use of individual in-depth interviews, which made it possible to gain deeper and more detailed insight into the experiences and attitudes of the health staff. The study also had some limitations. First, due to cost and time constraints, we visited only one province and one municipality out of 63 provinces/municipalities, which limits the generalizability of the findings. Second, there was no triangulation of data through use of other data collection methods, such as observation, to validate our findings. In this study the researchers were not permitted to interact with mothers. Therefore, comments about the mothers and reference to their experiences were from the provider perspective. Third, language was a barrier for direct communication between the researcher and interviewees [42]. Fourth, the interpreter was employed in the A&T offices, which could have created bias. The interpreter had a good understanding of the program, and thus translated the content well. However, using an interpreter affiliated with A&T might have made the participants less willing to disclose negative views about the training. While the inclusion of different staff groups working at different levels might enhance the transferability of findings, the apparent successful implementation of EENC in Viet Nam and lack of significant barriers reported suggest that the findings may have limited transferability to settings with more profound challenges in maternal and newborn care.

In conclusion, health staff reported improved staff and mother satisfaction, and health benefits for both the mothers and newborns from EENC after receiving training and coaching using the WHO approach. An approach to develop competencies, with a focus on practical training and coaching, should be promoted to form, reinforce and sustain recommended practices of health staff.

## Supplementary information

**Additional file 1.** Interview guide

## Data Availability

Deidentified transcribes are available from the last author on reasonable request.

## Abbreviations

WHO: World Health Organization
EENC: Early Essential Newborn Care
EIBF: early initiation of breastfeeding
UNICEF: United Nations Children’s Fund
A&T: Alive & Thrive
QoC: Quality of Care
NTP: Normalization Process Theory
