# Supplementary material for: Health staff experiences with the implementation of early essential newborn care guidelines in Da Nang municipality and Quang Nam province in Viet Nam
Source: BMC Health Serv Res. 2020 Jun 26;20:585. doi: 10.1186/s12913-020-05449-2 (PMC7318527; doi:10.1186/s12913-020-05449-2)
Supplement: Supplementary file 1 — Additional file 1. Interview guide [file 12913_2020_5449_MOESM1_ESM.pdf]

Appendix 1: Interview guide

Introduction for Interviews in Hospitals in Da Nang and Quang Nam:

Thank you for taking the time to talk to me today. Before we start I wanted to give you a brief introduction to why I am here. I am a Masters' student in Public Health Nutrition, from Oslo University College in Norway. For my Masters' Thesis, I am doing an assessment of the implementation of Early Essential Newborn Care, with main focus on breastfeeding. I will use the information I get from our talk today, to answer some of the questions in my master's thesis. This will be anonymous, which means that I will not mention your name in my assignment or what hospital you work in. If it is ok with you the interview will be recorded, so that it is easier for me to transcribe it later and get the information correct. These recordings will be deleted as soon as I hand in my assignment in May 2018.

Also, I want to let you know that if there are any questions you do not wish to answer because of personal reasons etc., please let me know, and we will skip those questions.

Does this sound ok?

Background:

1. How long have the interviewee been in his/her profession?
2. Can you tell me how a "normal day" at work is like?

The training in EENC:

1. What expectations did you have to what you were going to learn during the training?
2. Which of the topics that were reviewed during the training were especially relevant to you and the hospital you work in?
3. Do you feel that you have learned what you expected to learn from the training?
4. In your opinion, what could have been done differently to increase the learning outcome from the training?

The time after completed training:

1. In what way has the training influenced your practice of your profession?
2. Do you feel that it is challenging to perform what you learned during the training in practice?
    a. What is more challenging/why is this challenging?
    b. Are these challenges something you discuss with your colleagues?
3. Do you observe that there has been a change in practice/routines in your Unit/Hospital?
4. In what way does your workplace facilitate for full implementation of EENC?
    a. In what areas do you think that the facilitation can be improved?

EENC and breastfeeding:

1. First, I would like you to describe what happens when a child is born when it comes to Early Initiation of Breastfeeding – please, tell me something about your experience
2. Is this something new, after you attended the training?
    a. If so, what happened before?
3. What is your experiences with the mothers' reactions to Early Initiation of Breastfeeding?

4. In your opinion, does the mothers' have sufficient knowledge about exclusive breastfeeding and the benefits of this?
5. What measures are taken if the mother/child is unable to breastfeed?
6. What would you say is the general attitude towards exclusive breastfeeding in your community?
7. What do you think could improve exclusive breastfeeding among mothers in your community?
8. At this regard, did the EENC-training provide you with new knowledge that you can use to improve exclusive breastfeeding?

Benchmarks of EENC

1. Can you explain how the policy from the Ministry of Health was disseminated?
2. At this point, how is the EENC program financially supported?
   a. Do you get sufficient funding?
3. What is your experience related to how the key stakeholders react to the policy? Do they think that the policy is good/bad?

Health Facility EENC Standards

1. Can you please elaborate on how the training in EENC has influenced the policies and environment in your hospital?
   a. In your opinion, has the implementation of EENC been successful so far?
   b. Based on your own experiences, what can be done to make the implementation even more successful?
   c. Can you tell me something about the challenges you have experienced related to the implementation of EENC?
2. How are the strengths and areas of improvement addressed by the EENC hospital team in your hospital? Can you give me some examples? (How do they gather the data, what are the criteria's?)
3. What measures are being done to make sure that your hospital works to improve and sustain quality of care in the future?
4. Can you explain what is done if inappropriate clinical practices are identified?
   a. Do you experience that there are any differences in practice of EENC between the day shift and the night shift?
5. Is there a policy against promotion of baby food/ infant supplies in your hospital?
   a. How do you prevent that health staff does not promote this to the mother or other family members of the newborn?

Questions on Coverage Indicators for EENC

1. What challenges do you experience related to vaginal deliveries?
   a. In your opinion, what might be the solution to these challenges?
2. What challenges do you experience related to C-sectional deliveries?
   a. In your opinion, what might be the solution to these challenges?

Questions on Impact Indicators for Newborn Health

1. Can you explain how the data about EENC is collected?
   a. Do you experience that it is challenging to collect these data?
   b. Based on your personal experience with collection of data, do you have any suggestions to how these challenges could be solved?

2. Can you explain how the data you collect on EENC implementation is used to improve the program?
    a. Can you describe what measures is done if negative trends are identified?
3. In what ways do you feel that the reporting is helping your work?
4. At this point, are you collecting data on whether the mother is breastfeeding by discharge or not?
    a. Do you have a way of registering whether the baby has received any supplements apart from breastmilk before discharge?

Closing:

Is there anything you would like to add?
